# Comparison between Different Channel Coding Techniques for IEEE 802.11be within Factory Automation Scenarios

**DOI:** 10.3390/s21217209

**Published:** 2021-10-29

**Authors:** Lorenzo Fanari, Eneko Iradier, Iñigo Bilbao, Rufino Cabrera, Jon Montalban, Pablo Angueira

**Affiliations:** Department of Communication Engineering, University of the Basque Country (UPV/EHU), 48013 Bilbao, Spain; Eneko.Iradier@ehu.eus (E.I.); inigo.bilbao@ehu.eus (I.B.); rufinoreydel.cabrera@ehu.eus (R.C.); jon.montalban@ehu.eus (J.M.); pablo.angueira@ehu.eus (P.A.)

**Keywords:** IEEE 802.11be, PHY layer, channel coding techniques, factory automation, industrial wireless networks

## Abstract

This paper presents improvements in the physical layer reliability of the IEEE 802.11be standard. Most wireless system proposals do not fulfill the stringent requirements of Factory Automation use cases. The harsh propagation features of industrial environments usually require time retransmission techniques to guarantee link reliability. At the same time, retransmissions compromise latency. IEEE 802.11be, the upcoming WLAN standard, is being considered for Factory Automation (FA) communications. 802.11be addresses specifically latency and reliability difficulties, typical in the previous 802.11 standards. This paper evaluates different channel coding techniques potentially applicable in IEEE 802.11be. The methods suggested here are the following: WLAN LDPC, WLAN Convolutional Codes (CC), New Radio (NR) Polar, and Long Term Evolution (LTE)-based Turbo Codes. The tests consider an IEEE 802.11be prototype under the Additive White Gaussian Noise (AWGN) channel and industrial channel models. The results suggest that the best performing codes in factory automation cases are the WLAN LDPCs and New Radio Polar Codes.

## 1. Introduction

The automation of production processes is a central goal of the Industry 4.0 vision. Indeed, the use of modern technologies, such as wireless networks, would bring numerous advantages over the current wired networks. The most relevant benefits are the reduction of maintenance, installation, and network extension costs. The concept of automation and digitalization of the entire production process is known as Factory Automation (FA).

Intending to address the system features suitable for FA, over the years, both standardization committees [1,2,3], and main industry competitors have proposed several use cases [4,5,6,7]. Table 1 proposes a summary of the use cases proposed in Reference [2]. The use cases describe different levels of criticality as follows: Non-Critical, Critical, and Safety. Non-Critical consist of sensing and monitoring operations and other typologies of communications that do not have stringent requirements. Critical includes the closed-loop operations of the production processes where several factors, such as speed and temperature, have to be taken into account to prevent damages to personnel and equipment. Finally, Safety consists of Safety Integrated Systems that require high reliability and low latency to prevent any damage. The previous levels can be assessed by means of some metrics. Table 1 shows the following metrics: Packet Error Rate (PER) defines reliability, control cycle time, and payload length. The proposed parameters refer to the requirements of the MAC layer. The more critical the scenario, the more stringent are the requirements to be met.

One of the main challenges for wireless communications in FA is proposing a robust system that satisfies the low latency jointly to high-reliability requirements within short packet transmissions [8,9]. Such a result is achievable by a proper design of both MAC and PHY. At the PHY layer, the optimization of the access techniques and the choice of correct channel coding techniques are the way to go. In contrast, at the MAC layer, the non-determinism would guarantee, in particular, the achievement of the desired latencies. Regarding system robustness, many studies have proposed ad-hoc wireless network models to ensure continuity of service [10].

The MAC in IEEE 802.11 standards is usually non-deterministic, thus making them effectively incompatible with FA communication requirements. However, the enormous commercial success of the standard has encouraged the proposal of optimizations, particularly considering the IEEE 802.11b/n versions. Many of them focused on MAC layer modifications to provide time-aware scheduling and, consequently, reduce the latency performance. Some examples are RT-WiFi [11], IsoMAC [12], Priority MAC [13], or the recent HAR2D-Fi [14]. Other projects propose PHY and MAC joint optimization, as evidenced by the SHARP [15,16] and WirelessHP [9,17] projects, the latter being a standalone proposal. SHARP proposes at the PHY layer the following ideas: modifications on the IEEE 802.11g OFDM preamble and the use of CC codes. Regarding the MAC layer, the Time Division Multiple Access (TDMA) is the adopted technique. WirelessHP aims to offer a high-performing PHY layer for FA environments, taking the IEEE 802.11 PHY into account. The main changes include optimizing and reducing the preamble symbol and using CC and Reed-Solomon (RS) codes.

At the PHY layer, the optimization of IEEE 802.11 OFDM symbols has led to significant results, particularly on latency aspects [16,17]; on the other hand, the research related to the channel coding techniques concerning FA is not very comprehensive. In fact, in Reference [17], tests with RS and CC in industrial scenarios are proposed. However, for other candidates, there are neither tests nor simulations on industrial channels. Several possible candidates are presented in 5G-URLLC studies, among them LDPC, TURBO, and Polar codes. In particular, in Reference [18], the high reliability of these codes in AWGN is verified. In contrast, in Reference [19], it is demonstrated that, through parallel decoding, these codes can guarantee low decoding latencies, in fact not affecting the performance at the MAC layer.

This paper contributes to the studies related to IEEE 802.11be within industrial scenarios, offering a comparative analysis between different channel coding techniques for industrial implementations. The LDPC and CC codes presented here are within the IEEE 802.11ax standard, while TURBO and Polar are chosen for LTE and 5G NR, respectively. The analysis consists of simulations performed using MATLAB, considering the PHY layer proposed for IEEE 802.11be. The channels considered are AWGN and the industrial channels adopted by the IEEE 802.15.4 standard, i.e., CM7 and CM8, that reproduce Non-Line-of-Sight (NLOS) and Line-of-Sight (LOS) conditions inside the factories.

The structure of the paper is as follows. Section 2 focuses on the structure of IEEE 802.11be. Section 3 discusses the issues associated with the design of wireless systems for industrial environments. Section 4 presents the channel coding techniques proposed for IEEE 802.11be. Section 5 describes the simulation platform proposed in this work. Section 6 provides analysis and discussion of the simulation results. Finally, the conclusions are included in Section 7.

## 2. IEEE 802.11

The aim of this section is the description of the main PHY features of IEEE 802.11be. For this purpose, this part is composed of two subsections. Section 2.1 offers a brief overview of IEEE 802.11 standards, providing a state-of-the-art. Hence, after having described the main features of the PHY layer of IEEE 802.11be, the adopted PHY layer protocol is described, in Section 2.2.

### 2.1. State of the Art of IEEE 802.11

The IEEE 802.11 is a family of standards that specifies the PHY and MAC layers structure conceived for WLAN communications, initially developed for operating in the 2.4 GHz frequency band but now extended to the 5 and 6 GHz frequency bands.

The IEEE 802.11a offered several novelties regarding the PHY layer, which are then re-proposed in various IEEE 802.11 future amendments. It introduced the Orthogonal Frequency-Division Multiplexing (OFDM) technique for the 5 GHz operating frequency and CC as channel coding technique. IEEE 802.11g extended the usage of the IEEE 802.11a PHY layer in the 2.4 GHz [20]. Considering the channel bandwidth of 20 MHz, OFDM has been configured as follows: 64 tones, symbol length of 3.2 μs, and guard interval of 0.8 μs.

The IEEE 802.11n, developed for both 2.4 GHz and 5 GHz operating frequencies, introduced other features for the PHY layer. The main novelties are code rates of 5/6 and 40 MHz channel bandwidth. Furthermore, in IEEE 802.11n, the guard interval passes at 0.4 μs. Finally, LDPC appears, for the first time, as not mandatory for the 40 MHz channel bandwidth transmissions. In addition, IEEE 802.11ac offered further innovations, such as the opportunity to choose additional channel bandwidths, particularly 80 MHz and 160 MHz, and the option of 256-Quadrature Amplitude Modulation (QAM) for higher throughputs.

The latest IEEE 802.11ax standard was designed for low latency and MIMO communications and proposed significant changes in the PHY layer design. Among them, the introduction of the Orthogonal Frequency-Division Multiple Access (OFDMA) and the mandatory use of LDPC. OFDMA is applied in Multi-User MIMO in Uplink communications, while LDPC is the channel coding technique for channel bandwidths higher than 20 MHz. In comparison to OFDM, OFDMA allows multi-user transmissions by assigning a subset of subcarriers to different users, known as Resource Unit (RU). The use of this technique requires a new waveform structure compared to its predecessors. Indeed, the IEEE 802.11 ax shows a waveform formed by more subcarriers than the previous IEEE 802.11 standards [21].

The IEEE 802.11ax PHY layer concept is also followed in the standardization process of IEEE 802.11be [22]. The main challenges of this future standard involve the support of Real-Time Applications (RTA) and the coexistence between 3GPP technologies on the same unlicensed frequency bands. In Reference [21,23], the main proposals regarding the PHY layer are listed, which are studied and discussed by the different working groups: 4096 QAM modulation, 320 MHz bandwidth, MU-MIMO, and a new frame format. This new frame format will contain a preamble part dedicated to future features. Currently, the proposed format exhibits some issues related to short-frame transitions for its duration, which is longer than the previous releases [23]. However, IEEE 802.11be will include future enhancements for achieving lower latency performances in comparison to its predecessors [22].

### 2.2. IEEE 802.11be PPDU Format

**Figure 1:**
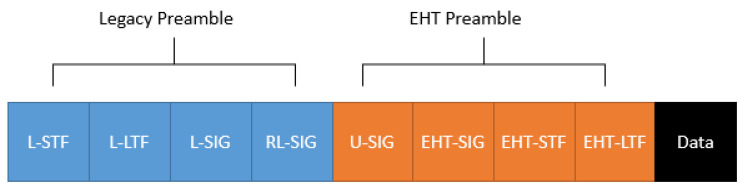
IEEE 802.11be PPDU format.

IEEE 802.11be will offer a new design of the physical layer convergence protocol data unit (PPDU) (Figure 1). The PPDU structure is splittable into four main blocks: Legacy Preamble, Extremely High Throughput (EHT) Preamble, and data.

This new standard will include backward compatibility, and, indeed, for this reason, the PPDU will contain the same legacy preamble structure introduced in IEEE 802.11a [23,24]. On the other hand, it will introduce the concept of forwarding compatibility in the IEEE 802.11 family. The long universal SIG (U-SIG) and the EHT-SIG will provide forwarding compatibility. U-SIG is composed of two OFDM symbols, defined as Version Dependent Information and Version Independent Information. The Version dependent Information will provide mechanisms similar to the HE signal field used in IEEE 802.11ax [23]. The following EHT-SIG field preamble will reserve future IEEE 802.11be features not included in the U-SIG, and it will be composed of common field and user-specific field, here information relevant for future communications, such as new channel coding techniques, will be added [23]. Finally, the short training field (EHT-STF) and long training field (EHT-LTF) preambles follow the same function as those offered in IEEE 802.11ax, i.e., fine-time and frequency tuning when using techniques, such as MIMO and OFDMA [23].

## 3. Discussion on Factory Automation Scenarios

This section reviews the main challenges of wireless communications for FA, examining the main characteristics of some of the channel models proposed in the literature. After discussing the main features of the channels, there is an analysis of the main problems inherent in designing wireless systems within industrial environments.

### 3.1. Proposed Industrial Channel Models

This paper studies the performance of the channel coding techniques within IEEE 802.11be, taking into account three different channel models (CM). One of them is the AWGN channel. The other two models are CM7 and CM8 and represent industrial environments. These models are based on the developments by Molisch et al. [25] and on the experimental results shown in Reference [26].

The CM7 is designed for Line of Sight (LOS) scenarios, while the CM8 describes non-LOS (NLOS) scenarios. These channels are both based on the Saleh-Valenzuela model. Furthermore, they represent environments for communications having lengths between 2 m and 8 m. Both models present a path loss model composed of free space path losses and shadowing components. The shadowing component is characterized by a log-normal distribution with zero mean and δ=6dB [27]. Both channels exhibit a coherence time of 30 ms. The coherence time of the channel is longer than the required E2E minimal latencies of the use cases addressed in this paper. Thus, re-transmission techniques, such as TDMA for reliability improvement, are not beneficial, as discussed in Reference [28,29].

### 3.2. Discussion on Factory Automation Wireless System Design Issues

As seen in Section 3.1, the CM7 and CM8 channels have a coherence time higher than the FA control cycle requirements; thus, the time-based re-transmission techniques do not provide the desired reliability effects [28]. Therefore, the first step to increase wireless systems’ reliability is to include more robust channel coding techniques.

Now, the discussion passes to the control cycle time issue. The Control cycle time defines when the master sends data to all sensors connected to the network. Considering that CM7 and CM8 describe environments close to 10 m × 10 m, it is possible to assume a network inside these areas, composed approximately of 20 slaves connected to an Access Point in the middle of the room, which is a commonly used configuration in these environments [29]. Therefore, considering a control cycle time of 10 ms, each slave must transmit 20 PPDU in a maximum time of 0.5 ms.

Hence, to ensure low latency values, it is necessary to send short data. As analyzed in several publications [18,30], there are specific channel coding techniques for transmitting short-sized data. The aim of these techniques is the simultaneous fulfillment of reliability and latency requirements. The latter condition is usually satisfied by the use of parallel decoding architectures.

## 4. Channel Coding Techniques

This section introduces the channel coding techniques considered potential candidates within the IEEE 802.11be standard regarding its application for FA scenarios. The candidates are the following: CC, LDPC, Polar, and Turbo codes.

The proposed CC and LDPC belong to the IEEE 802.11ax standard, while Polar and Turbo are the adopted channel coding techniques of the New Radio (NR) and LTE standards.

### 4.1. WLAN Convolutional Codes

CC was introduced by Elias in 1955 [31], offering an alternative to the first block codes. Compared with their counterpart, CC has a less complex structure, with several advantages in terms of latency and complexity that have guaranteed the success of these techniques for decades. Compared to block codes, the encoding/decoding consists of passing a stream of bits. Nevertheless, CC is not efficient with the first stream of data transmitted. For this reason, the encoder appends a known sequence to the transmitted data to overcome this problem. In the IEEE 802.11 standard, the sequence is known as tail-biting and consists of 6 bits set at 0.

Thus, tail-bits improve decoding performance, although this sequence provides an undesirable increase in the code rate [32].

### 4.2. WLAN LDPC Codes

LDPC codes were discovered by Gallager [33] in the 1960s but re-discovered in the late 1990s by Mackay and Davey [34]. Owing to their high performance, their application within numerous standards started in the 2000s. LDPC codes were first included in WLAN communication standards in 802.11n, and they were designed for operation modes in channel bandwidths higher than 20 MHz as an optional choice. From IEEE 802.11ax onwards, LDPC is mandatory in transmissions with bandwidths higher than 20 MHz [35].

In the IEEE802.11 standards, the LDPC family is the quasi-cyclic LDPC (QC-LDPC). It allows encoding a sequence of *K* bits in a block of *N* bits through a sparse matrix known as Parity Check Matrix (H). H has dimension N×N−K. Due to the sparsity property, it can represent LDPC with a bi-nodal graph known as Tanner Graph. This graph consists of *N* nodes, called variable nodes, and N−K nodes, known as check nodes. In the case of QC-LDPC, *H* presents a high sparsity. This property includes a secondary feature which is the Quasi Cyclicity. Quasi Ciclicity allows splitting *H* into several submatrices. From an application point of view, this allows full parallel decoding and low latency decoding.

### 4.3. LTE Turbo Codes

Turbo codes are among the first high decoding performance codes. They were proposed by Berrou et al. in 1993 [36]. Turbo coding consists of connecting two or more convolutional coders in parallel. Moreover, the usage of blocks allows increasing robustness in transmission. Among the different proposals, one of the best known is the version for the LTE standard [37], specifically for downlink communications through the Physical Downlink Shared Channel (PDSCH).

LTE Turbo model includes the parallel connection of two convolutional coders, four interleavers, a rate match, and a circular buffer. The position of the interleavers is the following: a first one between the two encoders, while the remaining ones correspond to the systematic bit sequence and the parity bits. The buffer allows correct decoding from the first transmitted frames, while the rate match adapts the encoded block to different code rates.

### 4.4. New Radio Polar Codes

Arikan introduced polar codes in the late 2000s [38]. From the beginning, they generated enormous interest in the scientific community for being considered the first techniques that can probably achieve the channel capacity. The high decoding performance offered in short packet transmission has allowed their inclusion within the 5G NR standard [39]. In particular, the Polar application includes uplink and downlink control information in enhanced mobile broadband (eMBB) communications.

The main feature of the Polar codes is the use of the channel Polarization technique. This technique is only appliable to the binary-input memoryless channel (B-DMC), also known as *W*, and is based on the decomposition of it. There are two categories of obtained channels, the perfect channels that are error-free and the noisy channels. The *K* information is transmitted through the perfect channels, while the *N*-*K* redundancy bits pass over the noisy ones. The noisy channel bits are known as Frozen bits, and they are a sequence of bits known to the receiver, typically set at 0. The generator matrix G defines this operation.

## 5. Proposed Channel Coding Platform for IEEE 802.11be

This section describes the structure of the IEEE 802.11be simulation platform. A high-level explanation of the architecture is given in Section 5.1, while Section 5.2 and Section 5.3, respectively, describe the proposed structure for encoding and decoding within the IEEE 802.11be. These modifications allow the inclusion of different candidates in the PHY layer. Finally, Section 5.4 discusses in detail the characteristics and settings of the proposed channel coding techniques, together with respective simulation parameters.

### 5.1. Platform Architecture

**Figure 2:**
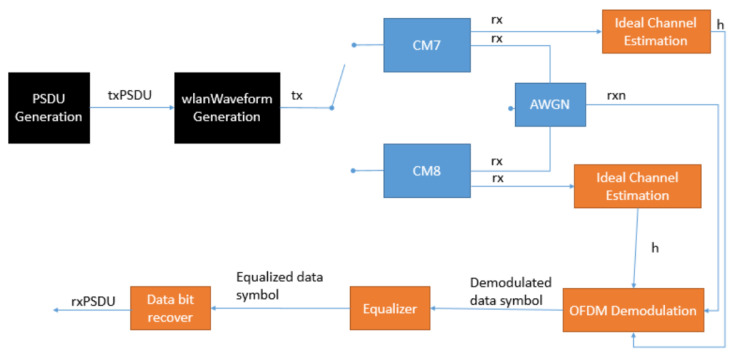
Block diagram model for reliability analysis.

The platform is based on the WLAN toolbox of MATLAB, and it offers the transmission of the IEEE802.11be waveform among two nodes. In Figure 2, the simulation steps are represented with a block diagram model. After the PSDU generation, there is its passage to the WLAN Waveform Generation block, where is generated the PPDU. The PSDU configuration requires the following parameters: PSDU length, Modulation Coding Scheme (MCS), guard interval, channel coding, and channel bandwidth.

Then, the obtained waveform, represented by tx in Figure 2, passes through the channel. In the simulator, three different channels are available: CM7, CM8, and AWGN.

On the receiver, the first steps consist of the OFDM demodulation of the received signal rxn and its equalization. Moreover, regarding CM7 and CM8 scenarios, the ideal channel estimation is applied before the OFDM demodulation. Then, the obtained symbols pass to the Bit Recovering block, which consists of two main phases: demapping and decoding.

### 5.2. Data Transmission Block

**Figure 3:**
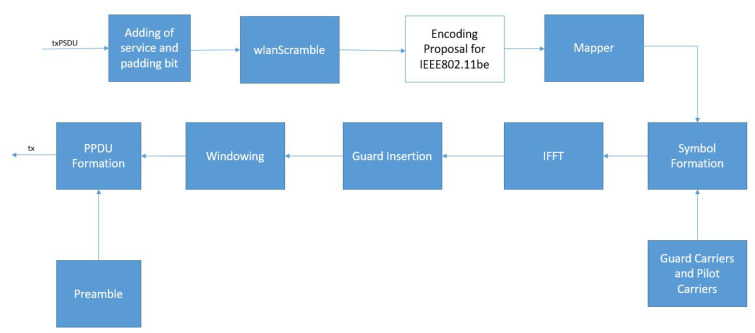
wlanWaveform structure.

The wlanWaveform generation block (Figure 3) generates the entire PPDU frame, which is composed of two parts: Preamble and Data. In this work, the encoding block, which is related to the generation of the PPDU data, has been modified to evaluate the candidates’ performance.

**Figure 4:**
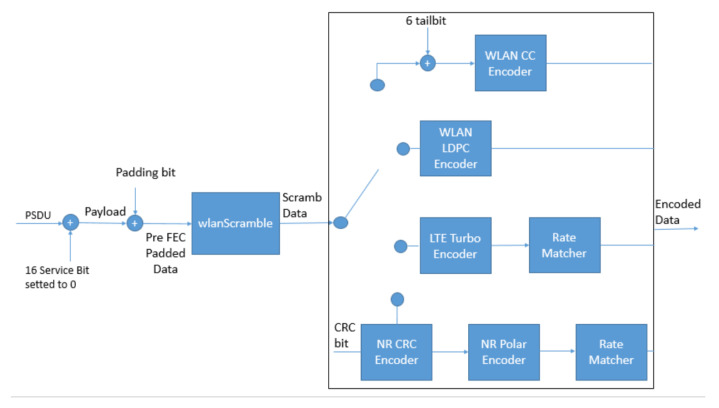
Modified block for studying the channel codes for IEEE 802.11be.

According to the configuration defined for the PSDU, the modified block offers the choice between four-channel coding techniques: WLAN CC, WLAN LDPC, LTE Turbo, and NR Polar. As shown in Figure 4, only the WLAN LDPC has a unique step in the encoding. In WLAN CC, a zero-bit sequence, defined tail bit sequence, is included in the data before the encoding. While concerning LTE Turbo, the encoded blocks have a code rate of 1/3, then a Rate Matcher block is added. This step is necessary for matching with the code rate defined in MCS. Finally, in NR Polar, the PSDU is concatenated with a CRC bit sequence before the encoding. Then, the obtained block passes to a Rate Matcher for the obtainment of the MCS code rate.

### 5.3. Data Bit Recover Block

**Figure 5:**
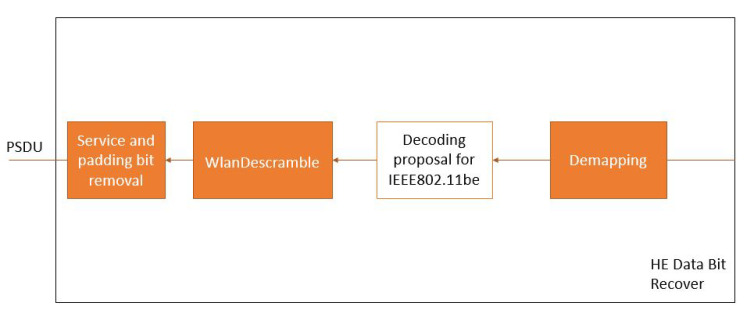
Data bit recover structure.

**Figure 6:**
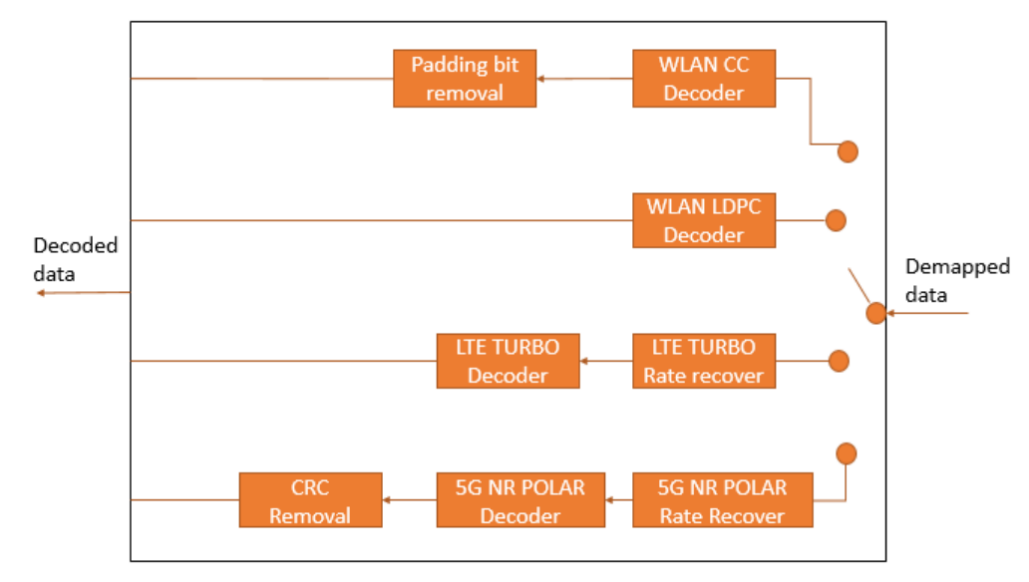
Proposed decoding structure.

The Data Bit Recover (Figure 5) recovers the PSDU from the OFDM symbols reserved for the data. This process consists of four main steps: demapping, decoding, descrambling, and removing the additional bits. Among these steps, this work proposes an adaptation of the IEEE 802.11 decoding block to analyze the channel coding techniques, as shown in Figure 6.

The changes applied to the decoding block allow the decoding of the candidate channel coding techniques. There are additional steps in the decoding for the WLAN CC, NR Polar, and LTE Turbo. In the case of WLAN CC, there is the padding bits removal after the decoding. In LTE Turbo, before the decoding, the blocks pass to a demapper block for matching with the decoder requirements. Finally, 5G NR Polar before the decoding has a Rate Recover step, while, after the decoding, there is the CRC Removal applied to the decoded block.

### 5.4. System Model Parameters

Table 2 summarizes the parameters used for the simulations. Regarding the data transmission within FA environments, these are the following configurations. Concerning PSDU, the adopted lengths are 32 B and 64 B, while regarding the MCS options, MCS0 and MCS1 have been chosen due to being the most robust configurations in IEEE 802.11 [21]. Both MCS present code rates 1/2, while the modulation used are BPSK for MCS0 and QPSK in the case of MCS1. Regarding the simulation setup, the following parameters have been adopted: the transmission of $10^{5}$ frames per point, BLER threshold of $10^{-3}$, and, finally, a simulation step between points of 0.25 dB.

The values of PER shown in Table 1 represent the desired reliability at the MAC layer, where, in addition to the effect of channel coding, there is the effect of the MAC layer techniques, such as the spatial retransmission techniques [28].

### 5.5. Channel Coding Configurations

Table 3 summarizes the chosen channel coding configurations. The WLAN CC encoder respects the design adopted by IEEE 802.11ax [40]. Both polynomial and tail-biting lengths are unchanged. Regarding decoding, the selected algorithm is soft decoding. The presence of the tail bit sequence reduces slightly the code rate. The effective code rate is defined as follows:

(1) $$R=\frac{PSDU}{2(PSDU+tb)},$$

where tb represents the length of the tail-biting sequence, and *R* represents the code rate.

In addition, WLAN LDPC respects the design for the IEEE 802.11ax standard, where are offered three different H models [40]. These models are selected according to the PSDU length and required code rate. In these simulations, the block length of 648 is used for PSDU of 32B and code rate 1/2, while the block length of 1296 for PSDU of 64B with code rate 1/2. Finally, for the decoding, the Belief Propagation algorithm is used with 12 iterations.

Regarding the 5G NR Polar, the proposed configuration ensures high decoding performance for short PSDU lengths. Such setup involves the concatenation between 5G NR Polar and Cyclic redundancy check codes (CRC). For decoding performance optimization, the CRC of length 11 bits is chosen for PSDU of 64B, while the CRC of size 24 bits type B, as proposed in Reference [41], is selected for PSDU of 32B. Regarding the decoding, the adopted algorithm is the CRC-aided successive-cancellation list (SCL). The length of the decoding list is 8.

Finally, for LTE Turbo, the rate match block is set to obtain an encoded block with a code rate of 1/2. The decoding algorithm is, in this case, Max-Log-MAP with 5 iterations, as in the standard [42].

## 6. Results

The section is composed of three parts. The first presents and discusses simulation results for an AWGN channel, the second discusses results for the CM7 channel, and, finally, the third gathers the plots for the CM8.

### 6.1. AWGN Results

**Figure 7:**
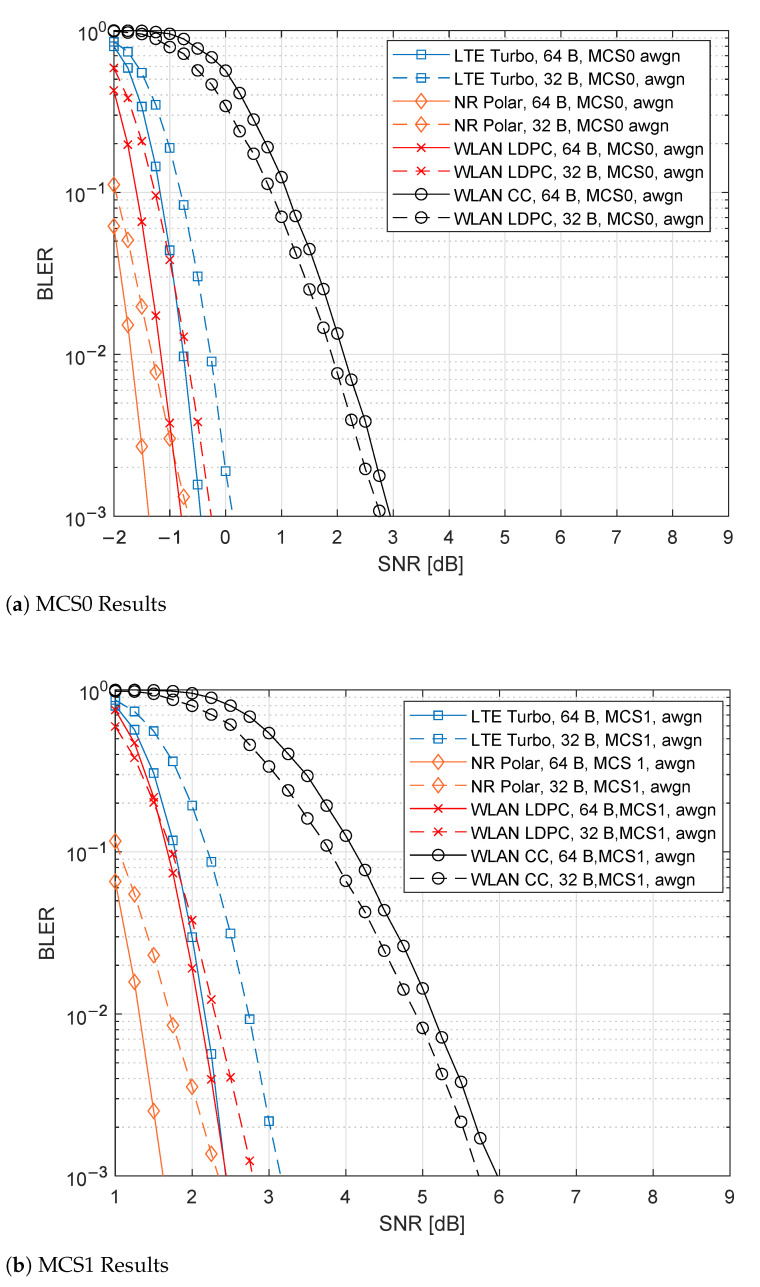
Comparison of the different channel coding techniques for AWGN channel.

Figure 7a gathers the results for MCS0, while Figure 7b includes those for MCS1. As observed in both figures, the first noticeable result is that the 5G NR Polar provides the best performance. In MCS0, they reach −1.5 dB per payload for PSDU of 64 B, while −0.6 dB for PSDU of 32 B. One other result observed in both figures is the WLAN CC behavior. The PSDU 32 B case offers noticeably better performance than the case with 64 B. An improvement close to 0.2 dB occurs. The presence of tail biting explains this, as shown in Equation (1).

Comparing the results for MCS0, WLAN LDPC offers very similar performance to 5G NR Polar. For both PSDU lengths, WLAN LDPC performs worst with a distance of 0.5 dB. In contrast, LTE Turbo distances itself by values close to 1 dB for 32 B, while, for 64 B, it is close to 0.5 dB. Finally, WLAN CC performs the worst. The distance to 5G NR Polar is close to 4 dB.

In the MCS1 case, WLAN LDPC and LTE TURBO provide a similar SNR value for 64 B, differencing of 1 dB respect the 5G NR Polar performance, while, for 32 B, WLAN LDPC is 0.5 dB away from 5G NR Polar, and LTE Turbo is 1 dB away. Finally, WLAN CC obtained the desired PER at almost 4 dB from the best-analyzed performance.

### 6.2. CM7 Results

**Figure 8:**
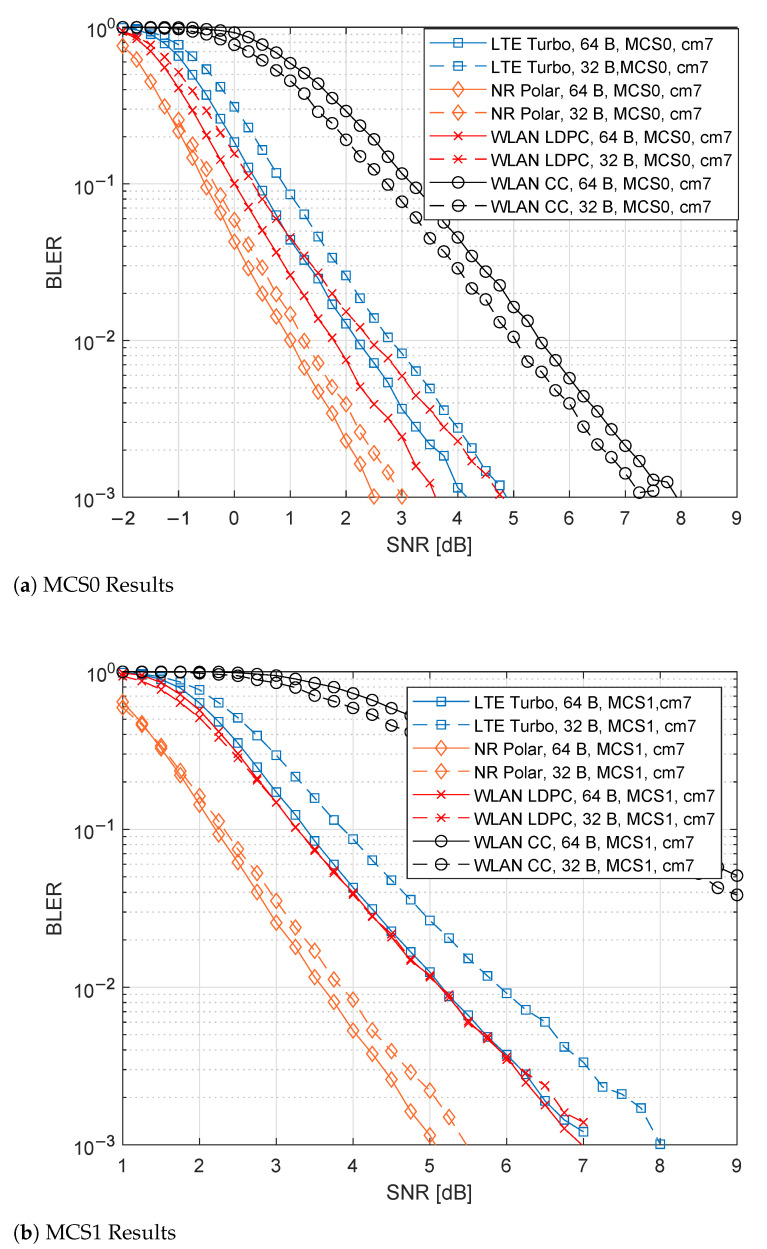
Comparison of the different channel coding techniques for CM7 channel.

Figure 8a,b show the results for MCS0 and MCS1, respectively. Comparing the results with Figure 7a,b, it is notable that results are worse for all the cases. In MCS0, 5G NR Polar are shifted to the right by about 4dB, as WLAN LDPC and LTE, while WLAN CC is about 5 dB worse. The trend is similar to the MC1 case.

For the MCS0, 5G NR Polar provides 2.5 dB for 64 B and 3 dB for 32 B. WLAN LDPC varies between 3.5 dB and 5 dB, respectively, for 64 B and 32 B. LTE Turbo varies between 4 dB and 5 dB. For WLAN CC, the SNR values are above 7 dB.

Regarding the MCS1, the 5G NR Polar performances for both the PSDU lengths reached values around 5 dB, while, in the case of WLAN LDPC, they pass around 7 dB. In the LTE Turbo simulations, the performance reached 7 dB and 8 dB, respectively, for 64 B and 32 B. Finally, for WLAN CC cases, the desired threshold is achievable for values higher than 9 dB.

### 6.3. CM8 Results

**Figure 9:**
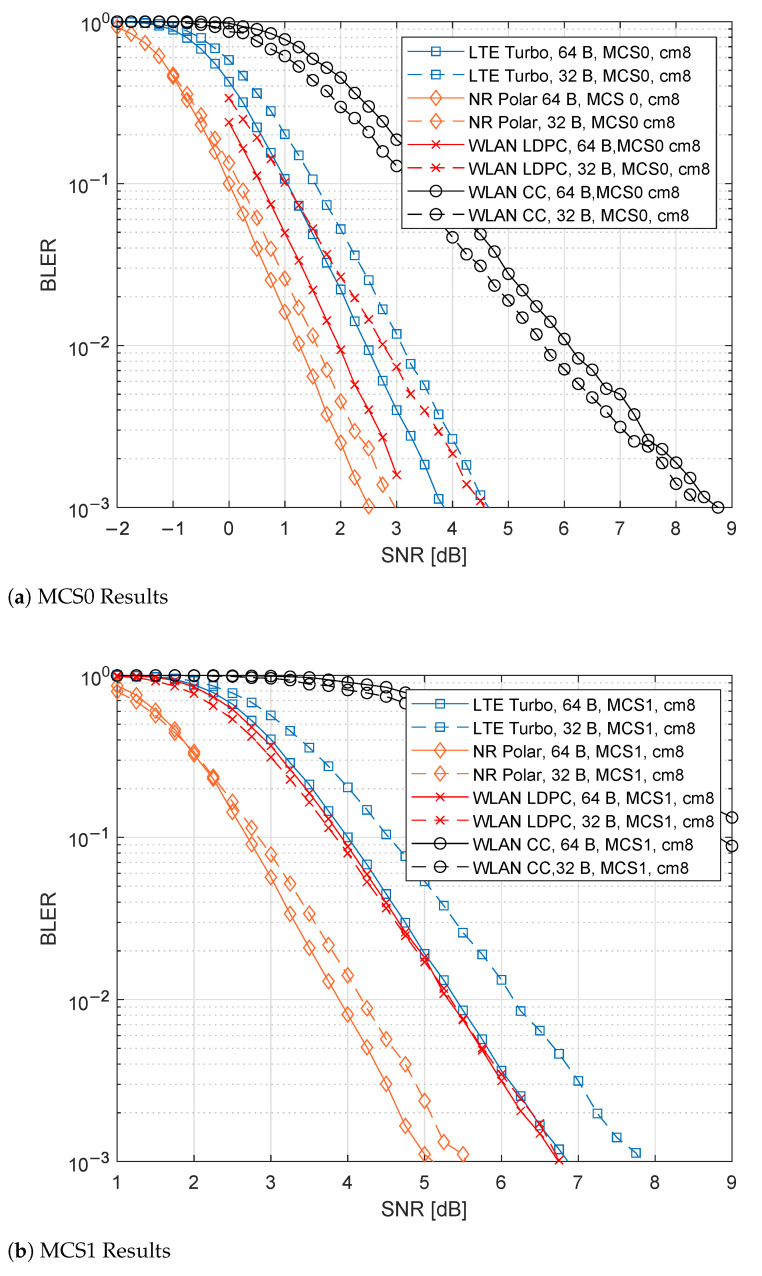
Comparison of the different channel coding techniques for CM8 channel.

In Figure 9a,b, the results for the MCS0 and MCS1 cases are presented, respectively. Compared to the AWGN case, the channel performance degradation is comparable to the CM7 case, with a 4-5 dB deterioration (see Section 6.2). Similarly, 5G NR Polar outperforms the other candidates for all the proposed configurations, while WLAN CC performs the worst. For the MCS0, 5G NR Polar reaches 2.5 dB and 3 dB, respectively, for the PSDU lengths 64 B and 32 B. Concerning MCS1, these performances pass to 5 dB and 5.5 dB. Regarding WLAN CC, the performances are close to 9 dB in the MCS0 configuration, while, for MCS1, such values are higher than 9 dB.

### 6.4. Discussion

Several channel coding techniques have been considered here as candidates for IEEE 802.11be in FA applications. The simulation platform adopted is based on the IEEE 802.11be PHY layer and includes different channel models. Analyzing the results, WLAN CC offers the worst performance, while WLAN LDPC and 5G NR Polar provide the best ones. Moreover, the degradation resulting from CM7 and CM8 simulations is higher than the other candidates. Indeed, WLAN CC reaches a degradation of 4 dB concerning AWGN simulations, while this difference is close to 2 dB for the rest of the candidates.

Considering these performances, WLAN LDPC and 5G NR Polar could be suitable for satisfying Safety and Critical use cases within IEEE 802.11be. Nevertheless, due to the demanding latency performance required by the FA use cases, this parameter needs also to be considered. Unfortunately, the proposed simulator only evaluates reliability, showing the BLER versus SNR plots results.

However, previous results offered by the literature help evaluate the latency performance by decoding latency results. This metric represents the time that the decoding processes require, considering the number of clock cycles required during such periods. Among the several models encountered in the literature, the choices are as follows. In the case of LDPC, Li’s model [43] proposes a BP decoder with full parallel decoding, while, in the Polar case, Fan’s model [44] applies an SCL decoder with semi-parallel decoding. Table 4 shows the properties of the decoders and the relative number of clock cycles, considering PSDU sizes of 32 B and 64 B.

*N* represents the block length, *R* the code rate, while *P* represents the number of updated nodes in the decoder. Considering the exclusive use of MCS0 and MCS1 in the proposed simulations, the only code rate applied is R=1/2.

Among the two candidates, LDPC, due to full parallel decoding, requires fewer clock cycles than Polar. In the =128B case, LDPC with BP at ten iterations needs five cycles, while, in the case of Polar, it reaches 32 cycles. In addition, for N=64B, LDPC with full parallel decoding needs five cycles, while Polar needs 16 cycles. Finally, it is necessary to highlight that the results show that LDPC provides the best decoding latency, but even the proposed Polar results can be considered suitable for the FA use cases.

## 7. Conclusions

This paper studies the performance of the IEEE 802.11 standard for FA scenarios. In particular, it is considered IEEE 802.11be, which is the first release of the IEEE 802.11 family designed to meet the requirements of FA use cases. The study compares different channel coding techniques within the PHY layer of the future standard, examining their decoding performance in different channel models. In general, WLAN LDPC and 5G NR Polar codes offer better performance than the other candidates. 5G NR Polar, in particular, appears as the best one in the BLER versus SNR plots. In addition, there is an analysis of the decoding latency of these two candidates. Indeed, latency is a demanding requirement in FA use cases. Concerning this analysis, WLAN LDPC results as the fastest one due to its full-parallel decoding property.

The main conclusion is that the LDPC codes designed for the IEEE 802.11 standards show suitable performance for being applied within IEEE 802.11be in FA scenarios, and, for this reason, IEEE 802.11be does not require changes regarding the adopted channel coding techniques. However, on the other hand, 5G NR Polar needs to be considered for its performance in future applicative scenarios within IEEE 802.11be, particularly for communications that require data sizes smaller than 32 B.

## Figures and Tables

**Table 1:** Factory automation use cases.

Use Case Category	PER	Control Cycle Time [ms]	Payload Length [B]	Applicative Example
Safety	$10^{-7}$	t ≤ 0.5	6–24	Alarms
Critical	$10^{-7}$	0.25 < t ≤ 12	8–1024	Close-loops controls
Non-Critical	$10^{-6}$	4 < t ≤ 50	12–33k	Monitoring operations

**Table 2:** Simulation parameters.

Parameters	Values
PSDU Lengths [B]	32, 64
Modulation	QPSK, BPSK
Code Rate	1/2
Channel Models	AWGN, CM7, CM8
Channel Parameters (AWGN is not included)	Mean = 0 dB, σ = 6 dB
BLER threshold	$10^{-3}$
Transmitted Frames	$10^{5}$
SNR steps [dB]	0.25

**Table 3:** Channel coding parameters.

Parameters	Encoding Details	Presence of Rate Matcher	Decoding Details
WLAN CC	Tail-bit sequence of 6 bit	No	Soft Decoding
WLAN LDPC	Considered blocks: 648, 1296	No	BP with 12 iterations
5G NR Polar	Considered 5G NR CRCs: 11 bit, 24 it type B	Yes	SCL with List size of 8
LTE Turbo	It follows the guidelines of the standard	Yes	Max-Log-MAP with 12 iterations

**Table 4:** Decoding algorithm clock cycles.

Parameters	LDPC	Polar
Decoding Algorithm	BP	SCL
Model	Li et al.	Fan et al.
Parallelism	Full	Semi
Required Clock Cycles	$\frac{NR}{P}$	$\frac{N}{P}log_{2}\frac{N}{4P}$
P	N	64
Iterations	10	no
Clock Cycles for N = 128 B	5	32
Clock Cycles for N = 64 B	5	16

## Data Availability

Not applicable.
